# Wide-ranging organic nitrogen diets of freshwater picocyanobacteria

**DOI:** 10.1093/ismejo/wrae236

**Published:** 2025-02-23

**Authors:** Elliot Druce, Stephen C Maberly, Patricia Sánchez-Baracaldo

**Affiliations:** School of Geographical Sciences, University of Bristol, University Road, Bristol BS8 1SS, United Kingdom; Lake Ecosystems Group, UK Centre for Ecology & Hydrology, Lancaster Environment Centre, Lancaster LA1 4AP, United Kingdom; School of Geographical Sciences, University of Bristol, University Road, Bristol BS8 1SS, United Kingdom

**Keywords:** picocyanobacteria, nitrogen metabolism, dissolved organic nitrogen, amino acid transporters, chitin, mixotroph

## Abstract

Freshwater picocyanobacteria (*Syn/Pro* clade) contribute substantially to the primary production of inland waters, especially when nitrogen is limiting or co-limiting. Nevertheless, they remain poorly understood ecologically and genomically, with research on their nitrogen acquisition mainly focused on inorganic sources. However, dissolved organic nitrogen is often a major component of the freshwater nitrogen pool and it is increasingly evident that many forms are bioavailable. Comparative genomic analyses, axenic growth assays, and proteomic analyses were used here to investigate organic nitrogen acquisition mechanisms in the *Syn/Pro* clade. Comparative analysis of the genomes of 295 freshwater and marine strains of picocyanobacteria identified a large diversity of amino acid transporters, the absence of degradation pathways for five amino acids (asparagine, phenylalanine, serine, tryptophan, and tyrosine), and alternative mechanisms for chitin assimilation (direct chitin catabolism vs initial acetylation to chitosan and subsequent degradation). Growth assays demonstrated the widespread bioavailability of amino acids, including basic amino acids though the known basic amino acid transporter is not encoded. This suggests further genetic components are involved, either through extracellular catabolism or the presence of novel transporters. Proteomic analysis demonstrates the dual utilization of nitrogen and carbon from the amino acid substrate and provides evidence for a mild stress response through the up-regulation of lysine biosynthesis and FtsH1, potentially caused by accumulation of secondary metabolites. Our results are relevant to understanding how picocyanobacteria have come to thrive in dissolved organic nitrogen-rich oligotrophic environments and explores how their different molecular capabilities may influence communities between habitats.

## Introduction

Picocyanobacteria (< 2 μm in diameter) represent the smallest group of Cyanobacteria, yet have a large impact on global aquatic ecosystems [1]. They thrive in a wide range of habitats [2, 3], where they are major primary producers [4, 5]. Globally warming temperatures are expected to promote picocyanobacterial growth due to their wide thermal tolerance, increasing their influence as primary producers [1]. Research into their ecology, evolution, and genomic capabilities has predominantly targeted marine environments [6–8]. However, picocyanobacteria frequently dominate freshwater cyanobacterial communities (primarily *Synechococcus* and *Cyanobium* spp.), contributing up to 90% of total lake cyanobacteria biomass [9] (with cyanobacteria often constituting a large proportion of total phytoplankton biomass [10]). This is commonly attributed to their large surface-area to volume ratio [11]; however, only recently have studies begun to address the question of ecological distribution and adaptation using genomic data, a framework that offers considerable insight into ecological processes [12–14].

Picocyanobacteria (also known as the monophyletic clade *Syn/Pro*) radiated within microcyanobacteria, a monophyletic clade containing lineages with small cell diameters (< 3 μm) [15]. The *Syn/Pro* initially comprised marine taxa, though subsequent sampling has improved the phylogenetic resolution, and four sub-clusters are now recognized [6]. Marine *Synechococcus* strains, prevalent throughout the global oceans, are found in sub-cluster 5.1 with 20 sub-clades further identified based on ecology and biogeography [16]. A sister group to sub-cluster 5.1 contains *Prochlorococcus*, split into high-light and low-light-dwelling clades [17]. Meanwhile, sub-clusters 5.2 and 5.3 contain picocyanobacteria from a greater diversity of habitats, encompassing fresh water, brackish, and marine strains. Widespread sampling has recently markedly expanded genomic information on freshwater picocyanobacteria, enabling greater elucidation of their adaptation to their environment, though no ecotypes are yet identified [13].

As sources of bioavailable nitrogen (N), more attention has been paid to inorganic N (e.g. ammonium [NH_4_^+^], nitrate [NO_3_^−^], and N_2_-fixation) than to dissolved organic N (DON). Recent studies have shown, however, that cyanobacteria are mixotrophs and can utilize DON, and specifically amino acids (AAs), as their N source [18, 19]. The DON pool is a heterogeneous mixture of nitrogenous compounds with significant concentrations of urea, free AAs, oligopeptides, nucleic acids, and humic substances amid many thousands of other, primarily uncharacterized, compounds, including chitin and glyphosate [20–23]. Chitin, one of the most abundant natural compounds [24], has been shown to be bioavailable to some cyanobacteria in its natural particulate form in addition to its DON form as chitosan [21, 22]. Likewise, the herbicide glyphosate is increasingly found in fresh waters and increasingly demonstrated to promote cyanobacterial growth [23]. DON can originate from a variety of allochthonous sources, including human and livestock excretion, cellular decay, soil leachate, and atmospheric deposition [20]. In inland waters, DON commonly represents the bulk of total dissolved N in oligo- and meso-trophic waterbodies, which picocyanobacteria tend to dominate [25–27]. Sixty percent of the total DON pool is thought to be readily metabolized by primary producers, significantly increasing known bioavailable N concentration, and contributing to available nutrient load [28, 29].

AAs are an essential bioavailable component of DON, found as both readily consumed dissolved free AAs or dissolved combined AAs that form variously sized polypeptides. The concentration of free AAs in surface waters is typically in the nM range, yet their rapid turnover and efficient microbial uptake suggests a disproportionately large contribution to N uptake [30, 31]. As a proportion of total DON, the pool of total dissolved AA (combined + free AAs) in fresh waters is 5–28% [32, 33], making up a greater proportion of DON than in marine environments: 1–12% [34]. Additionally, oligotrophic waterbodies have a greater proportion of DON vs total N than eutrophic waters [35], amplifying the contribution of AAs to facilitate N requirements in these low nutrient environments (compared to greater inorganic utilization in eutrophic habitats). The specific composition of DON is often varied and is generally characterized by the surrounding catchment and local land-use practices [36]. It is currently unknown how the specific composition of dissolved free AAs influences the proliferation of freshwater picocyanobacteria, though elucidating this would enable the prediction of picocyanobacterial communities based on watershed management and trophic status.

Understanding the role of DON in sustaining picocyanobacterial abundance in oligotrophic environments is essential for evaluating their mixotrophic capabilities. This study investigates the mixotrophic potential of freshwater picocyanobacteria and compares it to the better-studied marine picocyanobacteria. This involved firstly, a comparative genomic analysis to identify encoded assimilation capabilities of various DON compounds and differences based on habitat in 295 freshwater and marine picocyanobacteria strains. Secondly, growth assays of axenic cultures to determine if potential DON compounds could support growth. Thirdly, quantitative proteomic analysis of *Synechococcus* sp. CCAP1479/10 to investigate the intracellular response to growth on selected AAs as putative N sources. We find that mixotrophic potential is widespread in freshwater picocyanobacteria, potentially contributing to their growth in oligotrophic environments.

## Materials and methods

### Strains

Two freshwater (salinity <0.5 ppt) picocyanobacteria strains were obtained: *Synechococcus* sp. CCY9618 (Culture Collection Yerseke; isolated from Vinkeveen, The Netherlands) and *Synechococcus* sp. CCAP1479/10 (Culture Collection of Algae and Protozoa; isolated from Windermere, UK). Axenic cultures of these strains were produced using fluorescent-activated cell sorting (Supplementary Text S1).

### Taxa selection and genome datasets

Picocyanobacterial genomes (*Syn/Pro* clade) were obtained from the National Center for Biotechnology Information RefSeq database [37] in September 2023. The environment from which these strains were initially isolated was determined from the cyanobacterial metadata (e.g. Genbank, JGI, scientific literature). Genome completeness was assessed using BUSCO v5.6.1 [38], where genomes with a completeness score less than 90% (commonly held as the threshold for a high-quality draft genome [39, 40]) were excluded. A total of 295 high-quality cyanobacteria genomes were analysed, comprising 88 genomes from freshwater environments and 207 genomes from marine/brackish environments (Supplementary Table S1).

### Nitrogen assimilation gene identification

An in-depth search through the scientific literature and maps of metabolic pathways identified 328 genes involved in cyanobacteria N assimilation and AA biosynthesis/degradation. These searches identified experimentally characterized proteins involved in the transport, metabolism, and biosynthesis/degradation of N. In addition, KEGG [41] and MetaCyc [42] pathway mapping were utilized to identify putative pathways and enzymes involved in cyanobacterial AA biosynthesis and degradation. These target genes were used in comparative genomics analyses with selected query sequences (Supplementary Table S2).

### Comparative genomic analyses

Target genes in our genome dataset were identified using BLASTP v2.11.0+ [43]. An E-value threshold of 1 × 10^−5^ was used to return the best match per genome for each query sequence. Identified genes for each target were compiled and then aligned with MAFFT v7.520 [44] using local pair alignment. For each gene, phylogenetic trees were estimated in IQ-TREE v2.2.5 [45] using the -m MF option to determine the best model [46]. Homology of target genes were checked based on their phylogeny. The presence of target genes indicates the potential for functional capability in the strain, it does not guarantee functional activity.

### Phylogenomic analysis

Evolutionary relationships of the taxa utilized in this study were estimated using phylogenomic analysis. Our genome dataset comprised 295 picocyanobacteria genomes, plus eight *Synechococcus spongiarum* genomes (to complete the *Syn/Pro*), and an outgroup of 10 *Synechococcus elongatus* strains (Supplementary Table S3). Ortholog sequences from 143 protein-coding genes (based on previously published studies [47, 48]) were compiled from each genome of our expanded dataset for phylogenomic analysis, carried out following a previously published method (Supplementary Text S2) [14].

### Growth rate measurements

Axenic *Synechococcus* sp. CCY9618 and *Synechococcus* sp. CCAP1479/10 cultures were grown in 150 cm^2^ vented flasks containing 400 ml BG-11 media. After growth for 4 days at 10–20 μmol m^−2^ s^−1^ (spectral range 400 to 700 nm) from white LED light with a 16 h: 8 h light:dark cycle at 20°C, each culture was centrifuged for 5 min at 1260 × g and the pellet washed three times with N-free BG-11 medium [22]. The cultures were then cultivated for a further 24 h in 400 ml N-free BG-11 to remove residual N. Triplicate 25 cm^2^ vented culture flasks were prepared for each strain and N source with 11 ml of N-free BG-11 medium and 1 ml of culture inoculum, supplemented with a N source. N sources included organic (20 proteinogenic AAs, urea, chitin, glyphosate) and inorganic N (NH_4_^+^ and NO_3_^−^). These N substrates were selected based on their significant contribution to DON (AAs and urea [20]), their metabolic novelty (chitin [22] and glyphosate [23]), and their historically common use as N sources (NH_4_^+^ and NO_3_^−^ [15]). Two N concentrations were utilized. A high concentration (250 mg N L^−1^) based on the N content of BG-11 media, to compare growth on organic N substates to NO_3_^−^ in this commonly used medium for freshwater cyanobacteria in laboratory settings. A lower concentration (1 mg N L^−1^) was also utilized to improve the generalizability of the findings to ecological settings, using a more environmentally relevant N concentration to accurately mimic the concentrations encountered in freshwater natural environments [49–52]. Each flask was incubated for 14 days under the conditions described above. Picocyanobacterial growth was determined by daily measurement of optical density (OD) at 750 nm on 200 μl aliquots using a Multiskan SkyHigh Microplate Spectrophotometer (ThermoFisher Scientific, Waltham, MA, USA). Poor tyrosine solubility necessitated a reduced high N concentration of 25 mg N L^−1^ for this condition. Growth rates and lag phase duration were determined using Growthcurver v3.0.1 [53]. Statistical analysis was carried out using a two-tailed t test with FDR-adjusted *P* values (*Q*).

### Proteomic growth conditions

*Synechococcus* sp. CCAP1479/10 was selected for subsequent proteomic analysis based on its generally shortened lag phase on the tested organic N substrates and greater number of amino acid transporters (AATs) (N-II, N-III, GltS) compared to *Synechococcus* sp. CCY9618 (N-II, N-III). *Synechococcus* sp. CCAP1479/10 was grown and harvested as described above. Following N-free incubation, 1 ml of culture was inoculated into triplicate flasks containing 11 ml N-free BG-11 supplemented with 250 mg N L^−1^ of a N source (NO_3_^−^, arginine, asparagine, glutamate, proline) or no N for a total of six conditions. The selected organic substrates were chosen to include a range of chemical properties (charge) and preferred AAT substrates in this strain. Cultures were incubated for two to 5 days, until exponential phase was reached (48 h incubation for N-starvation condition), at 10–20 μmol m^−2^ s^−1^ white LED light with a 16 h: 8 h light:dark cycle at 20°C. 2 ml aliquots were subsequently collected for protein extraction.

### Protein extraction, quantitative proteomics, and data analysis

Protein content was extracted from each sample using a NoviPure Microbial Protein Kit (Qiagen, Hilden, Germany) according to the manufacturer's instructions. Protein concentration was determined using a Nanodrop Spectrophotometer 2000 (ThermoFisher Scientific, Waltham, MA, USA) and sent to the Proteomics Facility at the University of Bristol for quantitative proteomic analysis, see Supplementary Text S3 for details. Only proteins detected in all replicates were used for further analysis. ANOVA was used to determine significant enrichment among proteins, followed by Tukey’s Post-Hoc test (FDR-adjusted) to determine significance between conditions. Differentially expressed proteins (DEPs) were deemed statistically significant with a *Q* value less than 0.05 and a log_2_ fold change greater than 0.5/less than −0.5. Proteins were functionally annotated using eggnog (see Supplementary Figures S3 and S4) [54] and pathway enrichment analysis was carried out using KEGG [41] and hypergeometric distribution tests.

## Results

### Uptake capabilities of amino acids and other forms of DON

**Figure 1:**
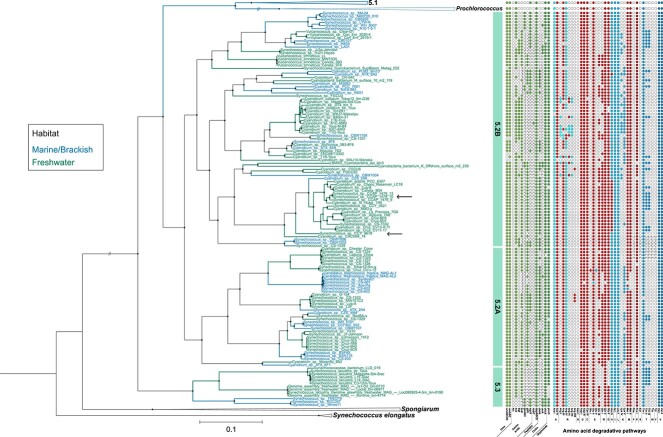
**Phylogeny of freshwater picocyanobacteria and encoding capacity for organic N uptake, assimilation, and AA degradation**. This maximal likelihood tree (IQ-tree v2.2.0) is based on 143 orthologous proteins, rooted using *Synechococcus elongatus* as an outgroup. Node support values were generated with ultrafast bootstrap approximations with bootstrap values over 50 displayed. Black nodes have support values of 100, green nodes have support values of 75, and red nodes have support values of 50. Each branch is colored based on the isolated habitat (see in-lay). Arrows indicate *Synechococcus* sp. CCAP1479/10 and *Synechococcus* sp. CCY9618, the two strains used for the subsequent experimental work. The sub-clusters within the *Syn/Pro* clade are labelled (5.2A/5.2B/5.3) with marine strains (5.1/*Prochlorococcus*) added for comparison. The presence or absence of organic N assimilation mechanisms is displayed, consisting of urea transport (*urtABCDE)* and metabolism (*ureABC)*, AATs (N-I (neutral), N-II (acidic), Bgt (basic), N-III (neutral), *gtrABC* and *gltS* (glutamate), and *agcS* (glycine)), peptide transporters (*oppC* (oligopeptides) and *dppABCD* (di-peptides)), chitin metabolism (chitosanase (*chdA* and *choA*) and chitinase (*chiA*) pathway, and glyphosate transport (*phnD*). Presence is indicated with green dots, with blank dots representing pathway absence. The presence and completion of AA degradation pathways is also shown. These pathway numbers are based on MetaCyc. Full pathways are shown with red dots and indicate full known functioning metabolic pathways for the selected substrate. Partial pathways are displayed with blue dots and indicate the presence of an initial catabolic enzyme but absence of one or more enzymes throughout the remaining metabolic pathway, suggesting potential routes for catabolism but a lack of understanding of the finer details of these specific pathways. Blank dots indicate the absence of the initial catabolic enzyme for that pathway.

There are seven AATs characterized in cyanobacteria, of which four are broad-substrate ABC-type transporters with varying substrate affinities and preferences: N-I and N-III for neutral non-polar AAs [55–58], N-II for acidic/neutral polar AAs [59, 60], and Bgt for basic AAs [59] (Supplementary Table S4). Though N-I is absent from the *Syn/Pro* clade, neutral AA uptake can occur via N-III which is encoded in 95% of freshwater picocyanobacteria (Fig. 1). In contrast, this neutral AAT is not as prevalent in marine picocyanobacteria: it is absent from the 5.1 and *Prochlorococcus* sub-clusters entirely and found only in marine *Synechococcus* of sub-cluster 5.2 (Supplementary Table S5). Our comparative genomic analysis indicates that the N-II transporter is more widespread: it is found in 95% of freshwater picocyanobacteria and 90% of non-*Prochlorococcus* marine picocyanobacteria (only present in 50% of *Prochlorococcus* strains) and represents the sole broad-substrate AAT among the majority of marine picocyanobacteria. The Bgt transporter is currently the only known active-uptake method for basic AAs in cyanobacteria [56]. However, this transporter is uncommon among the *Syn/Pro* clade, found only in sub-cluster 5.2 among freshwater (23%) and marine (18%) strains (almost all in sub-cluster 5.2B).

Additional AATs found in cyanobacteria are substrate-specific, predominately glutamate transporters reflecting the central role of this AA in N metabolism. Of the two sodium-dependent glutamate-specific transporters known, Gtr (a TRAP-type composed of three components: two integral membrane proteins (*gtrA* and *gtrB*), and a periplasmic binding domain (*gtrC*)) is present in marine picocyanobacteria across all sub-clusters, though *gtrC* (not essential for function [61]) is absent from marine strains, whereas GltS is found more commonly in sub-cluster 5.2, especially among freshwater strains (Fig. 1). However, these transporters are absent from the majority of picocyanobacteria, with Gtr only present in 25% of marine strains and GltS slightly more abundant, encoded by 49% of freshwater strains (Supplementary Table S5). In comparison, AgcS, a cyanobacterial glycine-specific transporter that has been expressed in *E.coli* [62], is prevalent in marine picocyanobacteria, especially among sub-cluster 5.1 (found in 99% of strains), and generally absent from freshwater strains (11% presence).

The uptake of other sources of organic N is also widespread among picocyanobacteria. Urea uptake through the Urt ABC transporter and urease activity is prevalent throughout the *Syn/Pro* (Fig. 1, Supplementary Table S5). Other common sources of DON include oligo- and di-peptides. The Opp oligopeptide transporter is not found in picocyanobacteria, however the Dpp di-peptide transporter is present in both freshwater and marine strains. The assimilation of chitin can take place through two pathways which are differentially encoded among picocyanobacterial sub-clusters (Fig. 1). Direct catabolism of chitin is more common in marine picocyanobacteria of sub-cluster 5.1, with 38% of these strains encoding chitinase (*chiA*) but lacking chitin deacetylase capability. In contrast, the potential of chitin acetylation into chitosan, and subsequent catabolism of chitosan with chitosanase, is found in sub-cluster 5.2 among both freshwater (56%) and marine (55%) strains with chitinase rarely encoded (7% of all sub-cluster 5.2 strains). Glyphosate is a novel source of organic N, with uptake enabled via the phosphonate transporter encoded by *phnD*. This is prevalent among most picocyanobacteria, only largely absent in freshwater strains of sub-cluster 5.3 (Fig. 1). However, it is important to recognize that the presence of these genes alone does not confer functional activity of these pathways, as seen in our growth assays below.

### Amino acid biosynthesis and degradation

Picocyanobacteria overwhelmingly have the capacity for AA biosynthesis, with almost a full complement of biosynthetic pathways found among all habitats and sub-clusters. The sole exception to this is the generation of alanine in *Prochlorococcus* (Supplementary Table S5). The absence of alanine dehydrogenase *(ald)* is found in the high-light and low-light I *Prochlorococcus* ecotypes, from which all known AATs are absent (Supplementary Table S5), suggesting an alternative alanine biosynthesis pathway or a requirement for extracellular alanine import through novel transporters.

Of the 61 AA degradation pathways analysed, 32 are identified in picocyanobacteria, either partially (encoding initial enzyme but lacking subsequent enzymes) or completely (Fig. 1). Of these 32 pathways, 29 are found in freshwater (and marine) picocyanobacteria with the remaining three (arginine asparagine (asparaginase) and glutamate (deamination and hydroxyglutarate) pathways found only in marine strains. Complete degradation pathways are found for nine AAs: alanine, arginine, aspartate (2 pathways), cysteine, glutamate (2 pathways), glutamine (2 pathways), glycine, methionine (2 pathways), and proline. Meanwhile five AAs: asparagine, phenylalanine, serine, tryptophan, and tyrosine, lack components of any degradative pathway in freshwater picocyanobacteria.

### Organic N bioavailability

**Figure 2:**
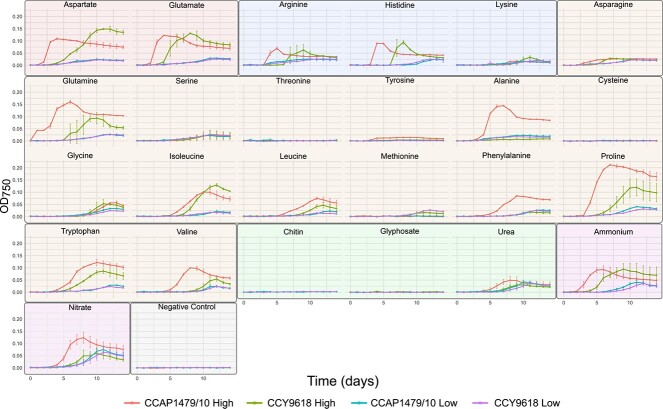
**Growth curves of *Synechococcus* sp. CCY9618 and *Synechococcus* sp. CCAP1479/10 cultivated under different N sources at different concentrations.** Substrates are highlighted based on type: Acidic AAs (red), basic AAs (blue), neutral AAs (orange), other organic substrates (green), inorganic substrates (purple), and the negative control (grey). A high concentration (250 mg N L^−1^ (25 mg N L^−1^ for tyrosine due to poor solubility)) and low concentration (1 mg N L^−1^) of substrate was investigated. Values are expressed as means ± SD, n = 3*.*

*Synechococcus* sp. CCY9618 and *Synechococcus* sp. CCAP1479/10 encode the N-II (acidic AAs) and N-III transporter (neutral non-polar AAs) but lack Bgt (basic AAs), suggesting that basic AAs would be unavailable unlike N-II and N-III substrates. Most of the tested substrates, including basic AAs, exhibited some degree of bioavailability and supported the growth of both axenic picocyanobacteria strains under both high (250 mg N L^−1^) and low (1 mg N L^−1^) concentrations (Fig. 2). However, two polar AAs, cysteine and threonine, did not support growth. Limited tyrosine bioavailability was demonstrated only for CCAP1479/10 at a high concentration, whereas methionine was utilized, to some extent, only by CCY9618. In contrast, glyphosate and chitin were unavailable (although growth at high chitin concentrations could not be quantified because of particulate occlusion of the spectrophotometer).

**Figure 3:**
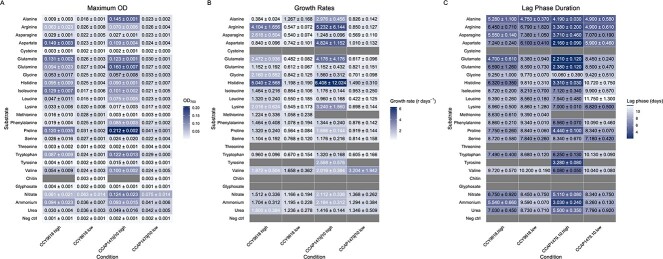
**Summary of growth characteristics for *Synechococcus* sp. CCY9618 and *Synechococcus* sp. CCAP1479/10 supplemented with nitrogen substrates at high concentration (250 mg N L^−1^) and low concentration (1 mg N L^−1^)**. (A) Maximum OD (OD_750_); (B) growth rate; (C) duration of lag phase. Values are expressed as means ± SD, n = 3. Note that the high concentration of tyrosine was reduced to 25 mg N L^−1^ due to poor solubility. Grey boxes indicate lack of growth or unquantifiable due to spectrophotometer distortion (high concentrations of chitin). * indicates a significant difference (q < .05) between species at high concentration. † indicates a significant difference (q < .05) within species between concentrations. Maximum growth rate and duration of lag phase was calculated using Growthcurver v3.0.1 [53].

Under high concentrations of N, the greatest picocyanobacteria yields occurred with aspartate for CCY9618 and proline for CCAP1479/10 (Fig. 3A). At the lower concentration of N, yield was greatest on NO_3_^−^, whereas the greatest yield on an organic substrate occurred with proline for both strains with yields of 49.4% for CCY9618 and 54.3% for CCAP1479/10 compared to NO_3_^−^ (100%) (Fig. 3A).

The fastest picocyanobacterial growth rates were associated with basic AAs (Fig. 3B). At a high N concentration, histidine supported the greatest growth rates for both CCY9618 (r = 5.04 ± 2.57 day^−1^) and CCAP1479/10 (r = 6.41 ± 12.02 day^−1^), however due to a single sharp increase in OD it is difficult to estimate rate accurately for this substrate. The greatest reliable growth rates utilized arginine as a N substrate for CCY9618 (r = 4.10 ± 1.66 day^−1^) and aspartate for CCAP1479/10 (r = 4.82 ± 1.15 day^−1^). At low concentrations, basic AAs also supported high growth rates, yet the greatest rates were achieved utilizing valine for both strains (CCY9618: r = 1.66 ± 0.36 day^−1^; CCAP1479/10: r = 3.20 ± 1.94 day^−1^).

### Picocyanobacterial lag phases and N concentration

Under a high N concentration, the shortest lag phases were found in CCAP1479/10, on substrates which can be immediately incorporated into N metabolic pathways—glutamate (2.21 ± 0.12 days) and glutamine (2.38 ± 0.12 days). Growth on aspartate also occurred with a short lag (2.16 ± 0.09 days), suggesting that acidic AAs may require minimal adaptation time. There are significant differences in the duration of lag phase between CCY9618 and CCAP1479/10 when grown under high and low concentrations of N (Fig. 3C). At high concentrations, growth on five AA substrates resulted in significantly shorter lag phases in CCAP1479/10 than in CCY9618 (aspartate (*P* adjusted value (*Q*) = 0.0049), histidine (*Q* = 0.014), valine (*Q* = 0.031), phenylalanine (*Q* = 0.0097), and proline (*Q* = 0.0073)) (Fig. 3C). In contrast, the lag phase was shorter on glycine for CCY9618 (*Q* = 0.022). At lower N concentrations, significant differences in the duration of lag phase were less prevalent (Fig. 3C).

### Proteomic response to growth on amino acids

TMT proteomics for CCAP1479/10 resulted in the identification of 5720 unique peptides and 1167 proteins. Of these, only proteins that were detected in all three biological replicates were analysed further, resulting in a total of 5134 peptides and 836 proteins (Supplementary Fig. S1 and Supplementary Table S6). FDR-adjusted ANOVA and Tukey Test analyses identified 224 unique DEPs (Supplementary Table S6). The 836 proteins detected in triplicate in this study correspond to 24.3% of the predicted 3441 proteins encoded in the CCAP1479/10 genome [14], consistent with percentages quantified from other studies, albeit towards the bottom of the expected range [63–67].

**Figure 4:**
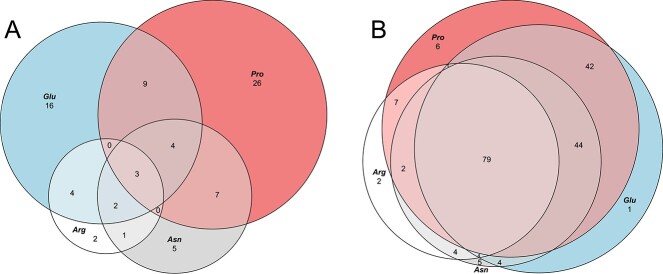
**Area-proportional Euler diagrams for amino acid nitrogen-substrate DEP overlaps compared to NO_3_^−^**. (A) Overlap of proteins up-regulated in *Synechococcus* sp. CCAP149/10 when grown under four AA conditions; (B) overlap of proteins down-regulated in *Synechococcus* sp. CCAP149/10 when grown under four AA conditions. For overlap of DEPs compared to N-starvation, see Supplementary Fig. S2.

The number of DEPs varied considerably among conditions. Of the 224 DEPs identified, 172 were linked to N-starvation and 160 were linked to growth across the four AA N-substrates compared to NO_3_^−^ (Supplementary Table S7). Compared to NO_3_^−^ and N-starvation, growth on glutamate (NO_3_^−^: 103 DEPs; N-starvation: 122 DEPs) and proline (NO_3_^−^: 112 DEPs; N-starvation: 116 DEPs) yielded more DEPs than growth on arginine (NO_3_^−^: 51 DEPs; N-starvation: 83 DEPs) and asparagine (NO_3_^−^: 69 DEPs; N-starvation: 90 DEPs). Of particular interest is the overlap of DEPs among AA substrate conditions. Only four up-regulated DEPs are shared between the four AAs and approximately half of all DEPs during growth with proline (42%) and glutamate (53%) are specific to that AA (Fig. 4A). In contrast, 79 DEPs were down-regulated among all four AAs compared to NO_3_^−^ (Fig. 4B).

### Pathway-enrichment

**Figure 5:**
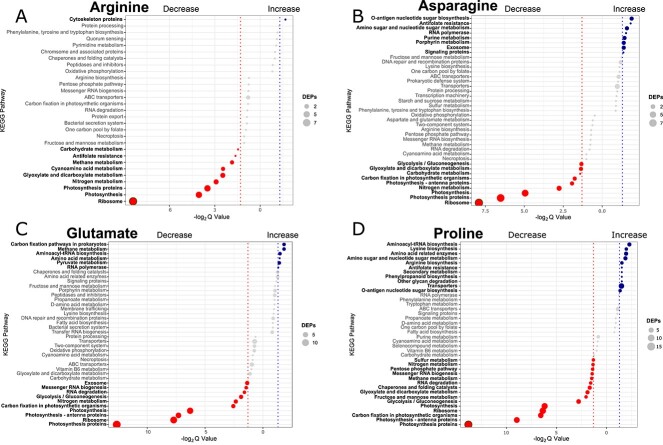
**KEGG pathway enrichment analysis of growth on amino acid nitrogen-substrate v. NO_3_^−^**. (A) Arginine v. NO_3_^−^; (B) asparagine v. NO_3_^−^; (C) glutamate v. NO_3_^−^; (D) Proline v. NO_3_^−^. Top 20 enriched pathways are shown with non-significant pathways in grey, significant pathways are in bold. The dashed lines indicate the significant thresholds of -log2 ± 1.5. See Supplementary Figure S5 for amino acid nitrogen-substrate v. nitrogen-starvation comparisons.

KEGG pathway-enrichment analysis identified 37 unique pathways with differential expression between growth on AAs and NO_3_^−^. Of these, 21 pathways are associated with over-expression under AA growth, though only five pathways are up-regulated under two or more AA-substrate growth conditions, indicating a large degree of variation in nutrient response (Fig. 5A–D). The four AA growth conditions display varying degrees of pathway enrichment, with arginine only significantly up-regulated in one pathway (“cytoskeleton proteins”) whereas growth on proline resulted in the significant up-regulation of 11 pathways (including “lysine biosynthesis” and “arginine biosynthesis”). Pathways involved with AA metabolism and transporters were expected to be up-regulated in AA-grown CCAP1479/10 compared to growth on NO_3_^−^, yet this was found only when grown with glutamate and proline (Fig. 5C–D).

### Nitrogen assimilation and amino acid associated DEPs

Compared to NO_3_^−^, AA metabolism/biosynthesis proteins involved with lysine (DapB, DapF, DapL), arginine (ArgJ, ArgB), and asparagine (GatC) were up-regulated during growth with at least one AA (Table 1). All except DapB were up-regulated under proline growth, with ArgJ and DapF up-regulated under both the proline and glutamate conditions. Conversely, the only N-assimilation associated protein up-regulated under asparagine growth is DapB, catalyzing an earlier step in lysine biosynthesis than DapF. Growth on arginine did not result in the up-regulation of any proteins associated with N assimilation, consistent with the lack of pathway enrichment. The abundance of Amt1 (NH_4_^+^ transporter) under proline growth is also identified, perhaps suggesting extracellular proline degradation and subsequent deamination forming NH_4_^+^. In contrast, the periplasmic substrate-binding component of the AAT N-III (NatI) is the only differentially expressed AAT subunit, down-regulated in CCAP1479/10 grown on glutamate compared to NO_3_^−^.

**Table 1:** **Selected DEPs in amino acid-grown *Synechococcus* sp. CCAP1479/10 compared to NO_3_^−^.** Tick indicates DEP in that AA N-substrate condition. Uncharacterized proteins were identified using BLASTP.

Function	Change	Protein	N source or AA metabolism pathway	Arginine	Asparagine	Glutamate	Proline
Nitrogen assimilation and amino acid metabolism	Increase	DapB	Lysine		✓		
	Increase	DapF	Lysine			✓	✓
	Increase	DapL	Lysine				✓
	Increase	ArgB	Arginine				✓
	Increase	ArgJ	Arginine			✓	✓
	Increase	Amt1	Ammonium				✓
	Increase	GatC	Asparagine				✓
	Increase	AroG	Aromatic AAs	✓	✓	✓	✓
	Increase	AccC	Leucine			✓	✓
	Decrease	GlyA	Serine/Glycine	✓			
	Decrease	GlnN	Nitrate	✓	✓	✓	
	Decrease	UrtA	Urea	✓	✓	✓	✓
	Decrease	NrtA	Nitrate	✓	✓	✓	✓
	Decrease	ThrC	Threonine			✓	✓
	Decrease	NatI	Amino Acids			✓	
Non-N transporters	Change	Protein	Name	Arginine	Asparagine	Glutamate	Proline
	Increase	SbpA	Sulfate-binding protein		✓		✓
	Increase	CmpA	Bicarbonate-binding protein				✓
	Increase	CmpC	Bicarbonate transport ATP-binding protein			✓	
	Increase	Ga0436389\_004\_46165\_47994	ABC transporter ATP-binding protein		✓	✓	✓
	Increase	Ga0436389\_026\_116581\_117309	LPS export ABC transporter ATP-binding protein		✓		✓
	Increase	Ga0436389\_030\_38087\_3984	ABC-F family ATP-binding cassette domain-containing protein				✓
Translation	Increase	GltX	Glutamate tRNA ligase		✓		✓
	Increase	AlaS	Alanine tRNA ligase			✓	✓
	Increase	LysS	Lysine tRNA ligase			✓	
	Increase	Rpl2	50S ribosomal protein L2				✓
	Increase	Rpl6	50S ribosomal protein L6				✓
	Increase	RplQ	50S ribosomal protein L17				✓
	Increase	RplR	50S ribosomal protein L18		✓	✓	
	Increase	RpsQ	30S ribosomal protein S17				✓
Stress Response	Increase	FtsH1	ATP-dependent metalloprotease FtsH1	✓	✓	✓	
Nucleotide Biosynthesis	Increase	PyrG	CTP synthase	✓		✓	
	Increase	PurH	Bifunctional purine biosynthesis protein purH		✓		✓
Respiration	Increase	NdhF1	Proton-translocating NADH-quinone dehydrogenase subunit F1 NdhF1	✓		✓	
Photosynthesis	Increase	AcsF	Magnesium-protoporphyrin IX monomethyl ester [oxidative] cyclase		✓	✓	
	Increase	CpcF	Phycocyanin alpha phycocyanobilin lyase CpcF			✓	✓
Transcription	Increase	RpoC1	DNA-directed RNA polymerase subunit beta'		✓	✓	✓
Carbohydrate Metabolism	Increase	ManC	Mannose-1-phosphate guanylyltransferase		✓		✓
	Increase	PpsA	Phosphoenolpyruvate synthase			✓	
	Increase	GlyP	Alpha-1,4 glucan phosphorylase				✓
Cell Cycle	Increase	MinD	Septum site-determining protein	✓			

### Transporters

In addition to NatI of the N-III system and Amt1, multiple other transporters, characterized and novel, increased in abundance when AA were provided as substrates (Table 1). Subunits of two systems are found among non-N associated DEPs. These include the substrate-binding protein of the sulphate ABC transporter (SbpA) (asparagine and proline vs NO_3_^−^) and subunits of the high-affinity bicarbonate ABC transporter – CmpC (ATPase; glutamate vs NO_3_^−^) and CmpA (substrate-binding protein; proline vs NO_3_^−^). Uncharacterized proteins associated with ABC transporters were also identified. In particular, Ga0436389_004_46165_47994 is up-regulated under asparagine, glutamate, and proline growth compared to NO_3_^−^ and is an unknown substrate ABC transporter ATP-binding protein. BLAST analysis of this protein reveals an MdlB domain superfamily involved in multidrug transport, primarily efflux, of small hydrophobic molecules [68]. This may suggest associations to hydrophobic AA export caused by build-up of intracellular AAs, though proline is the only hydrophobic AA tested in this proteomic analysis.

### Other proteins

Expression of proteins involved in multiple physiological processes were up-regulated in AA-grown vs NO_3_^—^ grown CCAP1479/10, including those involved with translation, photosynthesis, and stress response (Table 1). Translation-associated DEPs were identified on growth with asparagine, glutamate, and proline, but absent when grown on arginine. These proteins include tRNA ligases in addition to several core components of the 50S ribosomal subunit (Table 1). However, although the differential expression of tRNA ligases are limited to up-regulation, a substantial number of both 30S and 50S ribosomal proteins are down-regulated on AA substrates (Supplementary Table S7). This pattern also occurs with DEPs associated with photosynthesis. In comparison to NO_3_^−^, proteins involved with pigment biosynthesis (AcsF and CpcF) are up-regulated under asparagine, glutamate, and proline, whereas protein subunits of the PSI and PSII complexes are consistently down-regulated. Furthermore, FtsH1, linked to nutrient stress response in cyanobacteria, was up-regulated in the arginine, asparagine, and glutamate conditions, but not when grown on proline. However, FtsH1 was also up-regulated in the same AA conditions when compared to N-starvation (Supplementary Table S7).

## Discussion

The dominance of picocyanobacteria in oligotrophic environments has been mostly linked to reduced cell size and associated rapid nutrient uptake [69–71]. Other factors were first proposed in marine picocyanobacteria, with ecological genomics identifying genetic characteristics behind their oceanic distribution [7] and nutrient bioavailability, including their capacity for organic assimilation [8, 21, 72]. Although knowledge of freshwater picocyanobacteria is less developed [1], recent large-scale freshwater picocyanobacteria sampling [13] offers an opportunity to understand their genomic capabilities and mixotrophic potential, altering the paradigm of nutrient uptake for this fundamental keystone group.

### Diversity of amino acid bioavailability

Our growth assays on axenic cultures indicate that most AAs are potential N sources for freshwater picocyanobacteria. This contrasts with non-*Syn/Pro* freshwater cyanobacteria where AA utilization is variable and often limited (Supplementary Table S8), demonstrating that freshwater picocyanobacteria have among the most diverse DON assimilation potential. *S. elongatus* PCC 6301, a model cyanobacterium, has only been successfully grown on glutamine [73], whereas *Synechocystis* sp. PCC 6714 was limited to growth on glutamine, asparagine, and arginine, unable to utilize nine other AAs [73]. Heterocystous cyanobacteria also display a variety of capabilities, with *Pseudanabaena* spp. only able to grow on charged AAs [73] and *Anabaena* sp. PCC 7122 able to utilize neutral AAs but unable to grow on half of the AAs tested [74]. Only *Spirulina platensis* has similar uptake capabilities to those found here [75]. As such, the contribution of freshwater organic N diversity must be considered when examining picocyanobacterial abundance, providing an enhanced dietary supply in oligotrophic conditions. Meanwhile, although AA bioavailability is diverse and varied in picocyanobacteria, specific metabolic pathways could not be identified for a subset of AA substrates. This highlights the current lack of understanding of cyanobacterial AA metabolism outside of the central molecules (i.e. glutamate and aspartate), elucidation of which is necessary to achieve a holistic view of the cyanobacterial community response to nutrient diversity.

In addition to widespread bioavailability, the lag phase differences observed indicates the shorter adaptation time required for CCAP1479/10 for several substrates compared to CCY9618, though for some substrates this pattern was reversed. Differing microbial communities, catchment land use, and type of nutrient inputs can impact the AA composition, resulting in waterbodies with varying dominant total dissolved AA profiles [30, 49, 76]. Intraspecific variation in the adaptation time to individual nutrient sources may shape the initial microbial composition when first exposed to a nutrient flux, influencing community dynamics and dominant microbial strains. This may have implications for the wider cyanobacterial community, with the variety of species-specific responses to individual nutrient sources of heterogeneous DON potentially being a key driver in oligotrophic micro-community composition.

### Potential mechanisms for basic AA assimilation without dedicated transporters

Freshwater picocyanobacteria lack the basic AAT Bgt, though their capacity to utilize arginine and lysine as N sources highlights the complexity behind AA assimilation. We propose three possible mechanisms for basic AA uptake without a known dedicated transporter. The first is a broader specificity for the charged N-II AAT among the *Syn/Pro*, previously suggested in marine picocyanobacteria [7]. All AAT characterization has been carried out in non-*Syn/Pro* cyanobacteria [55, 56, 61], thus the uptake capacity within picocyanobacteria may be greater than expected. Secondly, an unidentified transporter may be responsible for basic AA uptake in picocyanobacteria, however, no such transporter was identified in the basic substrate condition of this study. Recent studies have identified putative AAT permeases in freshwater picocyanobacteria [13], though these remain uncharacterized with expression and uptake properties unknown. The knowledge gap regarding the molecular capabilities of freshwater picocyanobacteria is large, owing to the lack of a model organism and the absence of experimental research on this keystone group. Thirdly, AAs may be partially decoupled from AATs, with extracellular degradation bypassing the need for dedicated AATs and instead yielding available NH_4_^+^ or NO_3_^−^ for subsequent uptake. This extracellular AA oxidase activity has previously been demonstrated in various taxa, including cyanobacteria [77], diatoms [78], and green alga [79], though no up-regulation of AA oxidases were detected in this study. Two mechanisms behind extracellular AA degradation are known. The first involves the secretion of AA oxidases directly into the external environment, releasing NH_4_^+^ and H_2_O_2_, the latter of which acts as a cytotoxin [80]. The second mechanism is based on the passive diffusion of AAs into the periplasm through outer membrane porins, followed by extracellular catabolism through the action of cell surface AA oxidases [81]. Although oxidation rates are low and highly variable in aquatic environments [77], the importance of extracellular N release for picocyanobacterial N uptake remains to be clarified, with further work needed to identify the precise uptake mechanisms.

### Metabolic responses to growth on amino acids

The proteomic analysis of picocyanobacteria growth on AAs may indicate the initiation of a stress response and a reduced requirement for inorganic C. Lysine biosynthesis is up-regulated under most AA N-substrates tested, with lysine accumulation linked to environmental stress response throughout the biosphere [82–86]. These mechanisms are thought to involve an increase in lysine biosynthesis and subsequent conversion to various metabolites including saccharopine [84], cadaverine [87], and the compatible solute pipecolate [86], though DEPs associated with these were not identified in this study. Furthermore, an additional stress response protein (FtsH1) is up-regulated under arginine, asparagine, and glutamate-growth conditions. FtsH1 is involved in the cyanobacterial nutrient stress response, forming a FtsH1/3 protease complex which digests transcription factors repressing activation of Fe, P, N, and inorganic C assimilation proteins [88]. The conditions in this study provide an excess of nutrient, thus the up-regulation of nutrient stress responses compared to NO_3_^−^ is striking (Table 1). Although growth on some AAs equaled or exceeded that on NO_3_^−^, it is possible that accumulation of metabolites may have had negative consequences and been responsible for the stress response. In addition to this mild stress response, the proteomic analysis indicates that C skeletons from the AAs are being utilized and may explain the down-regulation of photosynthesis proteins. The molar Redfield ratio of C and N requirements (6.6:1) against those in glutamate and proline (5:1) are similar, which would facilitate balanced growth.

### DON assimilation mechanisms differ in freshwater and marine Picocyanobacteria

The diversity of AATs in freshwater picocyanobacteria is greater than in their marine counterparts. Whereas freshwater picocyanobacteria encode two broad-specificity AATs in addition to a glutamate-specific transporter, marine picocyanobacteria (predominantly sub-cluster 5.1/*Prochlorococcus* strains) encode N-II and the limited function of AgcS. These observed genotypic differences between freshwater and marine groups may be influenced by their respective evolutionary environments. For example, the composition of DON in marine environments is often more autochthonous than freshwater environments [89], decreasing nutrient profile heterogeneity and necessitating reduced AAT diversity. In addition, other factors such as temperature and salinity can influence the available fraction of DON, impacting the solubility and bioavailability of nitrogenous compounds [90]. The concentration of DON is consistently greater in fluvial and limnetic systems compared to the open ocean, with DON heterogeneity also increasing in fresh waters due to land use variation, land cover, and hydrology [36, 91–93]. This may promote the abundance of freshwater picocyanobacteria in their oligotrophic environments, where competition for the limited available nutrients may require greater diversity in nutrient uptake mechanisms. In contrast, the open ocean is less directly affected by anthropogenic influences and associated nutrient diversity, reducing the necessity of wide-ranging uptake capabilities. The prevalence of the N-II AAT in most picocyanobacteria may provide insights into the role of charged AAs. The preferred substrates for N-II are glutamate and aspartate, some of the most abundant AAs in freshwater and oceanic environments [94, 95]. This may provide a large bioavailable N source globally for the *Syn/Pro*, demonstrated by a high uptake rate of DON among marine environments [96].

**Figure 6:**
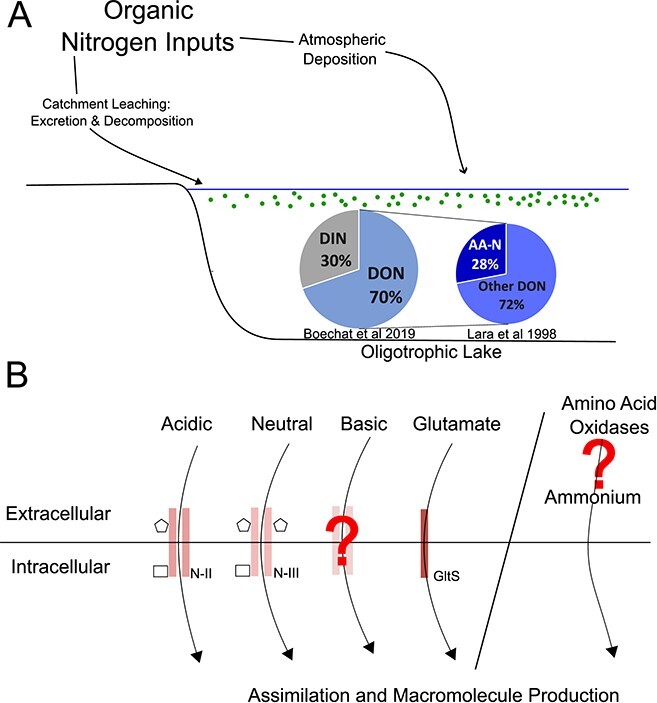
**Summary of picocyanobacteria AA-N diet.** (A) In oligotrophic environments, DON is the primary form of N [26]. Within the DON component, AAs make up a sizable proportion of bioavailable forms [33]. (B) Proposed mechanisms for AA uptake into the cell. The growth potential of AAs were proposed to be based on AAT preferences; however, this works finds that the capacity for AA-N based growth is greater than this hypothesis proposes. This suggests other factors are involved in determining AA utilization in picocyanobacteria, including novel transporters or extended transporter functions, and extracellular AA degradation.

This study utilizes comparative genomics to identify the organic N assimilation machinery in freshwater picocyanobacteria; however, it must be noted that there are limitations to this approach. The ability to express identified genes cannot be taken for granted, and the presence of assimilation-associated genes does not itself indicate that the functional activity is present. This has been previously seen in the freshwater picocyanobacterium *Vulcanococcus limneticus* LL, encoding the *nif* operon of N-fixation though yielding no evidence of its expression or capacity to fix N_2_ [12]. These issues can be addressed by use of -omics techniques to identify expression (though these have limitations themselves [97]), or the effective replication of true environmental conditions [98].

We find that AA bioavailability is widespread among freshwater picocyanobacteria. Freshwater picocyanobacteria thrive in low-nutrient environments where organic forms of N dominate (Fig. 6A) [27, 32, 99]. The broad range of AA bioavailability observed here may support the growth of picocyanobacteria in systems where the concentration of inorganic N is low. However, expected assimilation patterns based on encoded AATs are not identified, suggesting that AATs are not the only factors to be considered, and mechanisms for extracellular degradation (i.e. external oxidases) may be pivotal in DON utilization (Fig. 6B). In addition, potential mechanisms for organic N uptake (AA, chitin) differ between freshwater and marine picocyanobacteria, highlighting their adaptation to different ecological niches and the influence of the nutritionally heterogeneous nature of freshwater environments. Future research should elucidate the assimilation method of basic AAs and explore in greater detail the mechanisms and effective bioavailable concentrations for other organic N sources (i.e. chitin, glyphosate), including at lower concentrations which are present in oligotrophic environments. Research into organic nutrients is not limited to cyanobacteria—AAs are also bioavailable to freshwater algae [100]; however, the full diversity of response remains untested. Greater understanding of the association between nutrient inputs and community composition will enable future community changes to be predicted and encourage effective freshwater monitoring.

## Supplementary Material

Supplementary_Information_ismejo_wrae236

Supplementary_Tables_ismejo_wrae236

## Data Availability

The proteomics data have been deposited to the ProteomeXchange Consortium via the PRIDE partner repository with the dataset identifier PXD055938. The datasets generated and analysed during the current study are available from the corresponding author on request.
