# Supplementary material for: Wide-ranging organic nitrogen diets of freshwater picocyanobacteria
Source: ISME J. 2025 Feb 23;19(1):wrae236. doi: 10.1093/ismejo/wrae236 (PMC11851481; doi:10.1093/ismejo/wrae236)
Supplement: Supplementary_Information_ismejo_wrae236 [file supplementary_information_ismejo_wrae236.pdf]

Supplementary Information

Table of Contents

Supplementary Text

S1: Generation of Axenic Cultures 2

S2: Phylogenomic Analysis 3

S3: Quantitative Proteomics 4

References 6

Supplementary Figures

S1: Breakdown of TMT-quantified peptides in *Synechococcus* sp. CCAP1479/10 as a percentage of predicted number of total proteins for each COG category 7

S2: Area-proportional Euler diagrams for amino acid nitrogen-substrate differentially expressed protein overlaps compared to nitrogen-starvation 8

S3: Distribution of DEPs for sole AA-N growth compared to NO_3^- 9

S4: Distribution of DEPs for sole AA-N growth compared to N-starvation 10

S5: KEGG pathway enrichment analysis of growth on amino acid nitrogen-substrate vs. nitrogen-starvation 11

Supplementary Tables (located in excel document; WS = worksheet)

S1: Strains selected for comparative genomic analysis WS1

S2: Query Sequences for BLASTP analyses WS2

S3: Strains utilised for phylogenomic analyses WS3

S4: Amino acid transporter substrate preferences WS4

S5: Comparative genomics of N assimilation capabilities among the Syn/Pro clade WS5

S6: Summary of quantified proteins WS6

S7: *Synechococcus* sp. CCAP1479/10 DEPs grown on amino acid substrates v. NO_3^-/N-starvation WS7

S8: Amino acids as a sole nitrogen source in cyanobacteria WS8

S9: Common contaminants database WS9

Text

S1: Generation of Axenic Cultures

Two freshwater (salinity < 0.5 ppt) picocyanobacteria strains were obtained. One from Culture Collection Yerseke (The Netherlands) – *Synechococcus* sp. CCY9618 – and one from the Culture Collection of Algae and Protozoa (UK) – *Synechococcus* sp. CCAP1479/10. These strains were mono-phototrophic yet non-axenic. Strains were maintained in BG-11 media (Stanier, 1971) under 10 – 20 µmol m^{-2} s^{-1} of white LED light (in the spectral band of 400 - 700 nm) with a 16 h: 8 h light:dark cycle at 20 °C.

Fluorescent-Activated Cell Sorting

Fluorescent-activated cell sorting (FACS) was carried out on the *Synechococcus* strains to purify them from contaminating bacteria, based on previous research (Doppler, 2021). Samples were sent to the Flow Cytometry Facility in the School of Biomedical Sciences, University of Bristol. FACS sorting was performed using a BD Influx instrument (BD Biosciences, Franklin Lakes, NJ, USA). The optical configuration, in brief, employed 200 mW 488 nm blue (light scatter), 50 mW 640 nm red (chlorophyll detection), and 100 mW 552 nm yellow (chlorophyll excitation and detection) lasers. A small-particle light detector provided high sensitivity in detecting forward scatter and a 0.45 threshold on a logarithmic scale was used. A 4 mm obscuration bar was used for optimal forward scatter light detection with a 100 µm diameter ceramic nozzle tip and 21 PSI sheath pressure. Cells of interest were gated on using forward scatter v. side scatter, and singlets by light forward scatter v. trigger pulse width to exclude aggregated cells. Red chlorophyll positive events were identified by fluorescence emission from the 552 nm and 640 nm lasers using 670/30 nm bandpass filters. Cell sorting was achieved using single cell cloning precision into 96 well polystyrene plates.

Testing for Axenicity

Strains were tested for axenicity based on previous studies of the generation of axenic cyanobacteria from associated bacteria (1-5). After FACS, the resultant plates were incubated in 200 µL BG-11 medium per well under 10 - 20 µmol m^{-2} s^{-1} of white LED light with a 16 h: 8 h light:dark cycle at 20 °C. Once picocyanobacteria growth was visible (well turning green), each culture was streaked onto BG-11 agar plates and incubated for 15 days. Following this, the culture was transferred to 10 mL BG-11 medium and incubated for a further 15 days. Strains were then streaked onto LB and R2A agar plates and incubated for three days in darkness to assess bacterial growth. Samples from the LB and R2A agar plates were taken and observed under a microscope to examine the presence of contaminating bacteria. The plates were left to grow for a further 11 days in darkness, with plates inspected by eye at the end of this period. Plates without bacterial growth contained axenic cultures of *Synechococcus*. Axenic cultures were produced for both *Synechococcus* strains.

S2: Phylogenomic Analysis

To identify these ortholog sequences among our genome dataset, we performed BLAST searches (query sequences found in Supplementary Table S2) using BLASTP utilising an E-value cutoff of 10^{-5}. The hit with the highest score was retained and the corresponding protein sequences extracted. These sequences were then aligned using MAFFT v7.511 (-localpair, -maxiterate 1000 parameters) (Katoh, 2013) and maximum-likelihood trees constructed with IQ-tree v2.2.0 (Minh, 2020) (-m MF, -fast). These individual gene trees were used to identify 'true' ortholog sequences, determining these based on cluster association with the BLAST query sequences, with these true orthologs re-aligned with MAFFT. Re-alignments were inspected manually and mis-aligned columns were removed, as were alignment positions with a gap content higher than 80%. The model with the lowest Bayesian Information Criterion score was chosen as the optimal evolutionary model for each gene using IQ-TREE's -m MF option (Kalyaanamoorthy, 2017). Subsequently, a maximum-likelihood partitioned phylogenomic analysis was carried out with IQ-TREE, partitioned using the -p and -B 1000 (Hoang, 2017) parameters and the previously established evolutionary models with each gene assigned to its own partition. This analysis was repeated twice and the resulting trees compared to confirm there were no appreciable differences between them.

S3: Quantitative Proteomics

Protein Extraction and TMT-Labelling

Protein content was extracted from each sample using a NoviPure Microbial Protein Kit (Qiagen, Hilden, Germany) according to the manufacturer's instructions. Protein concentration was determined using a Nanodrop Spectrophotometer 2000 (Thermo Fisher Scientific, Waltham, MA, USA) and sent to the Proteomics Facility at the University of Bristol for proteomic analysis. 30 µg of each sample was reduced with tris(2-carboxyethyl)phosphine (10 mM) and incubated for one hour at 55 °C. Alkylation of cysteine residues was carried out with iodoacetamide (17 mM) and incubated for 30 minutes in darkness. Samples were finally digested with trypsin (1.25 µg) at 37 °C overnight. Samples were labelled with TMT 10Plex reagents according to the manufacturer's protocol (Thermo Fisher Scientific, Waltham, MA, USA) and the labelled samples were pooled together.

High pH Reverse-Phase Chromatography

An aliquot of the pooled sample (100 µg) was evaporated to dryness and resuspended in 5% formic acid. A SepPak cartridge was used to desalt the solution according to the manufacturer's instructions (Waters, Milford, MA, USA). The resultant eluate was prepared for fractionation by again evaporating to dryness before being resuspended in 20 mM ammonium hydroxide. Fractionation was carried out by high pH reverse-phase chromatography on an Ultimate 3000 liquid chromatography system (Thermo Fisher Scientific, Waltham, MA, USA). The sample was loaded onto an XBridge BEH C18 Column (130 Å, 3.5 µm, 2.1 mm x 150 mm; Waters, Milford, MA, USA) with peptides eluted over a steadily increasing gradient of 20 mM ammonium hydroxide in acetonitrile over one hour. The fractions produced were again evaporated to dryness and resuspended in formic acid (1%) to prepare for further fractionation.

Nano-Liquid Chromatography Mass Spectrometry

Further fractionation was carried out with nano-liquid chromatography mass spectrometry using an Orbitrap Fusion Tribrid mass spectrometer (Thermo Fisher Scientific, Waltham, MA, USA). Prepared peptides were loaded onto an Acclaim PepMap C18 nano-trap column (Thermo Fisher Scientific, Waltham, MA, USA) and washed with 0.1% (vol/vol) formic acid in 0.5% (vol/vol) acetonitrile. Peptides were then resolved on an Acclaim PepMap C18 reverse phase analytical column (250 mm x 75 µm; Thermo Fisher Scientific, Waltham, MA, USA) with a 150 min organic gradient. Seven gradient segments were utilised alongside Solvent A (0.1% formic acid) and Solvent B (SB; aqueous 80% acetonitrile in 0.1% formic acid) with a flow rate of 300 nL min^{-1}. The seven gradient segments included: 1-6% SB for 1 min; 6-15% SB over 58 min; 15-32% SB for 58 min; 32-40% SB for 5 min; 40-90% SB over 1 min then held at 90% SB for 6 min followed by reduction to 1% SB over 1 min.

Nano-electrospray ionisation (2.0 kV) produced peptide ions using a stainless-steel emitter with a capillary temperature of 275 °C and an internal diameter of 30 µm (Thermo Fisher Scientific, Waltham, MA, USA). An Orbitrap Fusion Tribrid mass spectrometer was used to obtain all spectra, controlled by Xcalibur 2.1 (Thermo Fisher Scientific, Waltham, MA, USA) and ran under an SPS-MS3 workflow in data-dependent acquisition mode. FTMS1 spectra were collected at a resolution of 120,000 with a maximum injection time of 50 ms and an automatic gain control target (AGC) of 200,000. Precursor ions were filtered with an intensity threshold of 5,000 and monoisotopic peak determination was set to peptide. A dynamic window (60 s +/- 10 ppm) removed previously analysed precursors. A quadrupole isolation

window (1.2 m/z) was used to isolate MS2 precursors. Resultant ITMS2 spectra were collected with a maximum injection time of 70 ms, AGC target of 10,000 and CID collision energy of 35%.

FTMS3 analysis was carried out at a resolution of 50,000, a maximum injection time of 105 ms, and an AGC target of 50,000. High energy collision dissociation at a normalised collision energy of 60% was used to fragment precursor ions and ensure optimal TMT reporter ion yield. The FTMS3 scan was able to include up to 10 MS2 fragment ions by enabling Synchronous Precursor Selection.

Data Analysis

Raw data files were quantified using Proteome Discoverer software v2.1 (Thermo Fisher Scientific, Waltham, MA, USA) and searched against the *Synechococcus* sp. CCAP1479/10 protein database (3,441 sequences) using the SEQUEST HT algorithm (Gold, 2015). Precursor mass tolerance was set to 10 ppm and MS/MS tolerance set to 0.6 Da. Variable modifications (oxidation of methionine (+15.995 Da), acetylation of protein N-terminus (+42.011 Da), methionine loss + N-terminus acetylation (-89.03 Da)) and fixed modifications (carbamidomethylation of cysteine (+57.021 Da) and addition of TMT mass tag (+229.163 Da)) were included as search criteria. A threshold of 2 missed cleavages was set and full tryptic digestion searches were performed with reverse database search enabled. The data was filtered using a 5% false discovery rate cut-off and contaminants were identified against a 'common contaminants' database (Supplementary Table S9). Peptide data was normalised to the total peptide in each sample and scaled using a pooled sample common to all runs. Statistical analysis was conducted using R.

References

1. Doppler P, Kriechbaum R, Singer B, Spadiut O. Make microalgal cultures axenic again - a fast and simple workflow utilizing fluorescence-activated cell sorting. *J Microbiol Methods*. 2021;186:106256.

2. Gao S, Kong Y, Yu J, et al. Isolation of axenic cyanobacterium and the promoting effect of associated bacterium on axenic cyanobacterium. *BMC Biotechnol*. 2020;20(1):61.

3. Han AW, Oh KH, Jheong WH, Cho YC. Establishment of an axenic culture of microcystin-producing *Microcystis aeruginosa* isolated from a Korean reservoir. *J Microbiol Biotechnol*. 2010;20(7):1152-1155.

4. Bolch, C.J.S., Blackburn, S.I. Isolation and purification of Australian isolates of the toxic cyanobacterium *Microcystis aeruginosa* Kütz. *J Appl Phycol.* 1996;**8:**5–13.

5. Vázquez-Martínez G, Rodriguez MH, Hernández-Hernández F, Ibarra JE. Strategy to obtain axenic cultures from field-collected samples of the cyanobacterium Phormidium animalis. *J Microbiol Methods*. 2004;57(1):115-121

Supplementary Figures

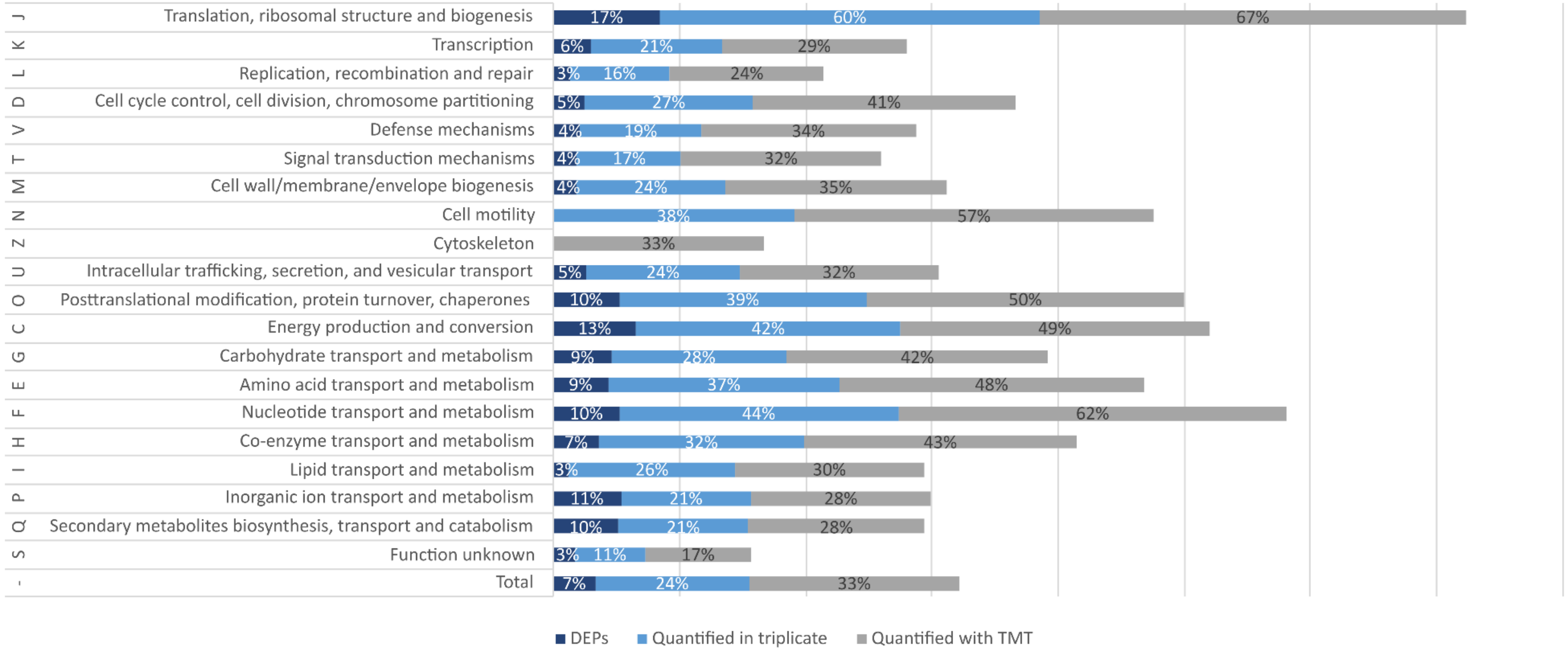

Supplementary Figure S1: Breakdown of TMT-quantified peptides in *Synechococcus* sp. CCAP1479/10 as a percentage of predicted number of total proteins for each COG category. Proteins identified with TMT proteomics (grey); proteins identified in triplicate (light blue); proteins identified as DEPs (dark blue).

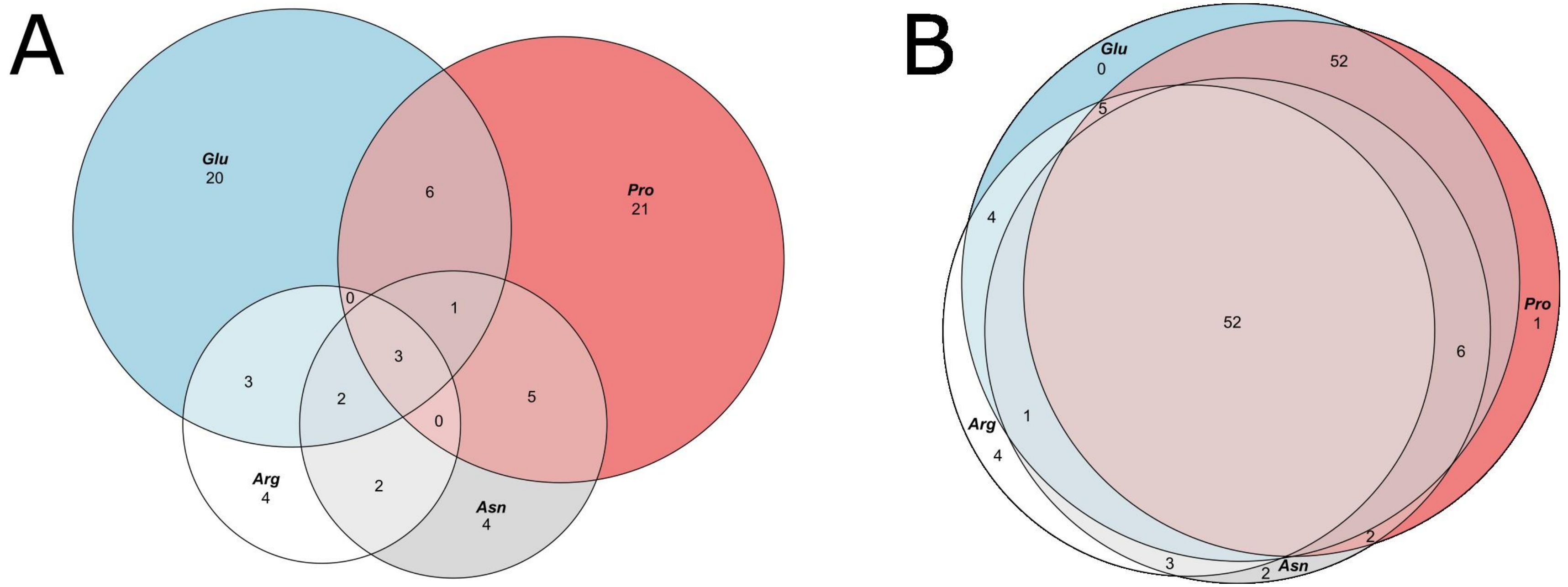

Supplementary Figure S2: Area-proportional Euler diagrams for amino acid nitrogen-substrate differentially expressed protein overlaps compared to nitrogen-starvation. A) Overlap of proteins up-regulated in *Synechococcus* sp. CCAP149/10 when grown under four AA conditions; B) Overlap of proteins down-regulated in *Synechococcus* sp. CCAP149/10 when grown under four AA conditions.

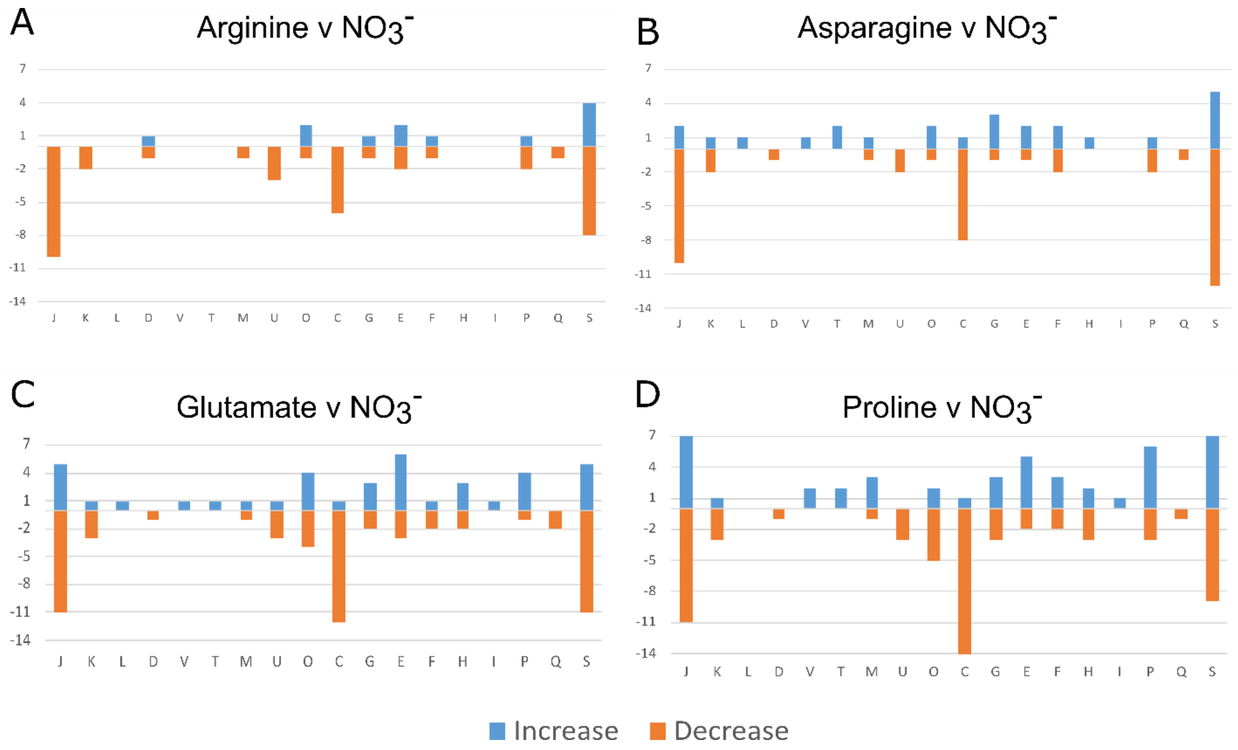

Supplementary Figure S3: Distribution of DEPs for sole AA-N growth compared to NO_3^-. Blue columns indicate up-regulated proteins, orange columns indicate down-regulated proteins, when grown on the specified AA. COG categories with no DEPs are not displayed. J: Translation; K: Transcription; L: Replication and repair; D: Cell cycle control and mitosis; V: Defence mechanisms; T: Signal transduction mechanisms; M: Cell wall/membrane/envelope biogenesis; U: Intracellular trafficking, secretion, and vesicular transport; O: Posttranslational modification, protein turnover, chaperones; C: Energy production and conversion; G: Carbohydrate transport and metabolism; E: Amino acid transport and metabolism; F: Nucleotide transport and metabolism; H: Coenzyme transport and metabolism; I: Lipid transport and metabolism; P: Inorganic ion transport and metabolism; Q: Secondary metabolites biosynthesis, transport and catabolism; S: Function unknown.

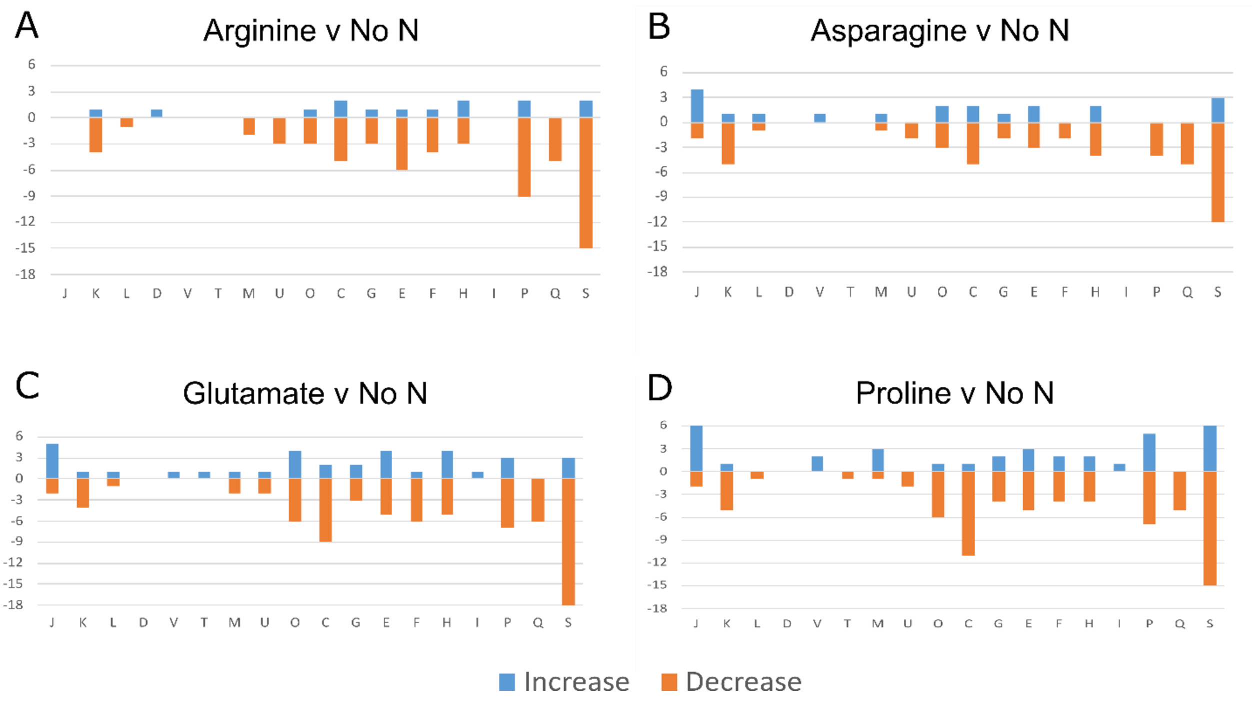

Supplementary Figure S4: Distribution of DEPs for sole AA-N growth compared to N-starvation. Blue columns indicate up-regulated proteins, orange columns indicate down-regulated proteins, when grown on the specified AA. COG categories with no DEPs are not displayed. J: Translation; K: Transcription; L: Replication and repair; D: Cell cycle control and mitosis; V: Defence mechanisms; T: Signal transduction mechanisms; M: Cell wall/membrane/envelope biogenesis; U: Intracellular trafficking, secretion, and vesicular transport; O: Posttranslational modification, protein turnover, chaperones; C: Energy production and conversion; G: Carbohydrate transport and metabolism; E: Amino acid transport and metabolism; F: Nucleotide transport and metabolism; H: Coenzyme transport and metabolism; I: Lipid transport and metabolism; P: Inorganic ion transport and metabolism; Q: Secondary metabolites biosynthesis, transport and catabolism; S: Function unknown.

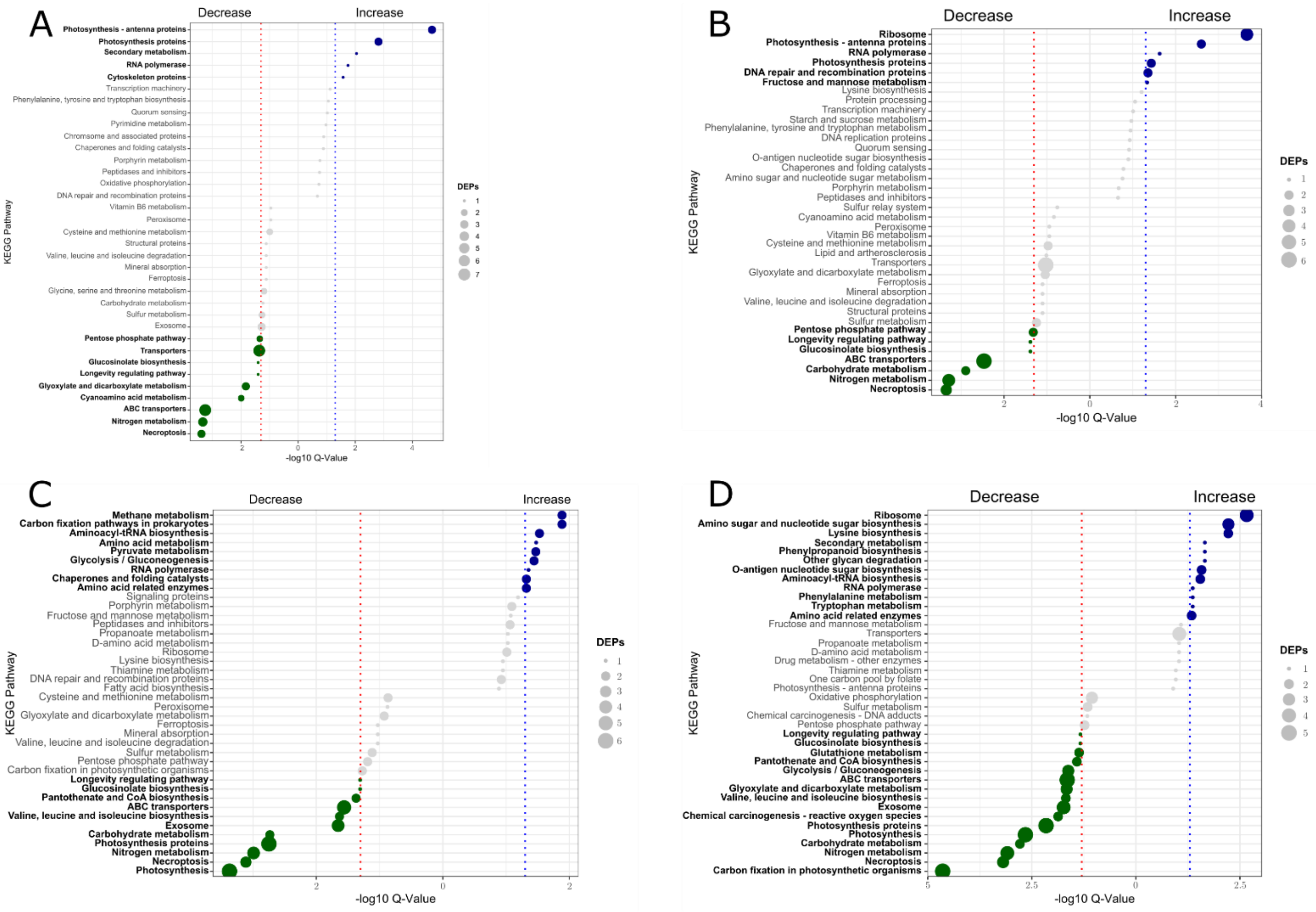

Supplementary Figure S5: KEGG pathway enrichment analysis of growth on amino acid nitrogen-substrate v. nitrogen-starvation. A) Arginine v. N-starvation; B) Asparagine v. N-starvation; C) Glutamate v. N-starvation; D) Proline v. N-starvation. Top 20 enriched pathways are shown with non-significant pathways in grey, significant pathways are in bold. Blue indicates up-regulated pathways while green indicates down-regulated pathways compared to N-starvation.
